# Developing and Validating a Digital Literacy Scale for Young Children Aged 3–6: Associations with Problematic Media Use

**DOI:** 10.3390/bs16101839

**Published:** 2026-10-08

**Authors:** Song Shi, Jiaman Wang

**Affiliations:** 1 School of Educational Sciences, Nantong University, Nantong 226019, China; liuchang861012@163.com; 2 School of Educational Sciences, Kashgar University, Kashgar 844000, China

**Keywords:** digital literacy, scale development and validation, problematic media use, young children

## Abstract

This study developed and evaluated a parent-report digital literacy scale for children aged 3–6 years and examined its associations with problematic media use. Theoretical analysis, interviews with experts and parents, and content validity assessment yielded 24 items. Independent samples of 398 and 603 parents from two locations in eastern China were used for exploratory and confirmatory factor analyses, respectively. The results supported four dimensions: digital operation and exploration, digital understanding and transfer, digital expression and interaction, and digital rules and risk prevention. The scale showed satisfactory internal consistency and initial evidence of construct validity and measurement invariance across child sex, place of residence, and age groups. The total score and all four dimensions were negatively correlated with problematic media use. After adjustment for demographic characteristics and the other dimensions, the three nonoperational dimensions remained negatively associated with problematic media use, whereas the coefficient for digital operation and exploration was not significant. The scale provides an initial research tool for assessing parent-reported digital competencies in young children. The observed associations do not establish that improving digital literacy reduces problematic media use.

## 1. Introduction

Young children encounter digital media in everyday family activities (Mannell et al., 2024; Schmid et al., 2025). As mobile devices, educational applications, and interactive digital tools enter the home, the ways in which young children access information, gain experience, and express ideas are changing (Liu et al., 2024; Marsh et al., 2017). For young children, digital literacy extends beyond device operation to understanding content, using media for learning and expression, and developing an awareness of rules and a capacity for reflection with adult support (Kumpulainen et al., 2020; Neumann et al., 2017). Adult support can help children participate in interactive, expressive, and creative digital practices; without it, their media use may remain largely limited to passive viewing or low-interaction exposure to content (Marsh, 2016; Sweetser et al., 2012). Assessing early digital literacy in children aged 3–6 years can therefore help describe how they participate in digital activities at home.

Existing frameworks commonly emphasize competencies related to information access and evaluation, online communication and collaboration, digital content creation, safety and protection, and problem solving (Tinmaz et al., 2022; Vuorikari et al., 2022). However, most existing digital literacy measures have been designed for university students, secondary school students, teachers, or parents and assume that individuals engage in digital activities relatively independently (Avinç & Doğan, 2024; Aydin et al., 2024; Jia et al., 2025; Tzafilkou et al., 2022). For young children aged 3–6 years, digital literacy does not primarily involve independently retrieving information, evaluating the credibility of complex information, or producing advanced digital content. Assessment instead needs to account for adult assistance and focus on behaviors within children’s everyday experience (Masoumi & Bourbour, 2024; Su & Yang, 2024). Research in early childhood education also indicates that the meaning and practice of young children’s digital literacy need to be interpreted in relation to age, family support, and specific everyday contexts (Soyoof et al., 2024). Measures and indicators are therefore needed that draw on general digital literacy frameworks while reflecting the characteristics of young children’s everyday digital behavior.

Existing approaches to measuring young children’s digital literacy have mainly followed two paths: quantitative questionnaire-based assessment and qualitative development of conceptual frameworks. Large-sample studies in China have used parent reports to examine young children’s digital literacy, multimodal practices, and developmental patterns in device-specific literacies. These studies have also examined their associations with family factors (Cao et al., 2024; Dong et al., 2022). These measures, however, have tended to focus on device use, operational experience, and home digital resources. Their coverage of digital content understanding, transfer of experience, expression and interaction, rule awareness, and risk prevention has been relatively limited. Research in South Korea distinguished operational digital literacy from cultural digital literacy and examined the related roles of home digital resources, maternal verbal interaction, and children’s learning behaviors (Moon, 2025; Moon & Shin, 2025). However, this two-factor structure may not fully capture the multidimensional nature of young children’s digital literacy. Cao et al. (2026) subsequently developed a locally grounded framework of early digital literacy in China through a grounded theory analysis of interviews with young children, parents, and teachers. However, the model was derived primarily from a small qualitative sample and has not yet received large-sample quantitative validation. Taken together, the measures and frameworks reviewed here leave gaps in dimensional coverage or quantitative validation for children aged 3–6 years. These gaps motivated the development of a multidimensional parent-report scale based on observable everyday behavior.

Previous research has linked early digital literacy to children’s age and residential context (Cao et al., 2024; Dong et al., 2022). Evidence of sex differences is more specific: Cao et al. (2024) found differences only in television literacy. These findings support examining whether a common scale measures digital literacy comparably across demographic groups. Measurement invariance testing evaluates the equivalence of measurement properties and provides a basis for meaningful group comparisons (Putnick & Bornstein, 2016). Accordingly, this study examined invariance across child sex, urban–rural residence, and age groups to assess the scale’s comparability across these populations.

Validation of a digital literacy scale for young children requires not only evidence regarding internal structure but also evidence based on relations with theoretically relevant external variables (Clark & Watson, 2019; Flake et al., 2017). Problematic media use does not simply refer to excessive screen time. It includes dysregulated features such as difficulty controlling use, emotional dependence, difficulty transitioning away from media, and disruption of everyday functioning (Domoff et al., 2019; Selak et al., 2025). In the present study, problematic media use was treated as a distinct construct and included as a theoretically relevant external variable to examine whether the new scale reflected individual differences in young children’s adaptive media engagement. The digital rules and risk prevention dimension includes boundaries for use, transitions away from media, and appropriate media-related behavior. This dimension was therefore expected to be negatively associated with problematic media use (Shawcroft et al., 2023). Associations involving the other dimensions may also vary according to media content and context, quality of participation, and adult support (Mallawaarachchi et al., 2024; Soyoof et al., 2024). Including problematic media use thus provides validity evidence based on relations with an external variable.

Accordingly, this study aimed to develop and validate the Digital Literacy Scale for Young Children for children aged 3–6 years. It also aimed to examine the scale’s internal structure, reliability, measurement invariance, and associations with problematic media use. The home is a central setting for young children’s digital media activities and digital literacy practices. A parent-report format was therefore used to draw on parents’ ongoing observations of everyday digital behavior at home and support developmental and contextual appropriateness (Diamond & Squires, 1993; Soyoof et al., 2024).

## 2. Scale Development and Validation: A Four-Phase Procedure

A four-phase procedure was used to develop and validate the scale. Phase 1 combined theoretical analysis, interviews with experts and parents, and expert evaluation to define the dimensions and generate initial items. Phase 2 used item analysis and exploratory factor analysis to screen the items. Phase 3 used confirmatory factor analysis to evaluate the scale structure and examined reliability, convergent validity, discriminant validity, and measurement invariance. Phase 4 examined associations between scale scores and problematic media use to obtain validity evidence based on relations with an external variable. Parents rated behaviors observed in their children’s everyday digital activities at home.

The Ethics Review Committee of the School of Educational Science, Nantong University, approved the study before participant recruitment and data collection (Approval No. NT202502; 12 February 2025). Written informed consent was obtained from the legal guardians of all participating children before the study began. Participation was voluntary, and participants had the right to withdraw at any time. Anonymity and confidentiality were maintained throughout the study.

### 2.1. Phase 1: Dimensional Framework, Item Generation, and Expert Content Validation

#### 2.1.1. Dimensional Framework

International policy frameworks and digital literacy research generally conceptualize digital literacy as a multidimensional set of competencies encompassing the use of digital technologies, content understanding and meaning making, digital participation and creative expression, and safety, protection, and responsible use (Nascimbeni & Vosloo, 2019; OECD, 2023; United Nations Committee on the Rights of the Child, 2021; Vuorikari et al., 2022). Research on digital competence in early childhood also emphasizes that young children’s digital literacy should be understood in relation to their developmental level, everyday experience, and adult support (Kontkanen et al., 2023; Masoumi & Bourbour, 2024). On this basis, digital literacy in young children aged 3–6 years was defined as comprising four dimensions: digital operation and exploration, digital understanding and transfer, digital expression and interaction, and digital rules and risk prevention.

Digital operation and exploration drew primarily on the OECD’s discussion of young children’s access to, use of, and understanding of digital technologies, together with early childhood research describing how children accumulate digital experience through familiarity, experimentation, and use. The dimension refers to young children’s recognition, exploration, and basic use of common digital devices (Masoumi & Bourbour, 2024; OECD, 2023). Digital understanding and transfer was informed by the UNICEF framework for children’s digital literacy, particularly its emphasis on information access, content understanding, and meaning making. It refers to understanding digital content with adult support and connecting digital experiences with everyday contexts (Kumpulainen et al., 2020; Nascimbeni & Vosloo, 2019). Digital expression and interaction drew on the United Nations Committee on the Rights of the Child’s emphasis on children’s opportunities to learn, express themselves, participate, and communicate in digital environments, together with the view that young children can be consumers, communicators, and producers of digital content. It was operationalized as the ability to use multimodal resources, including images, audio, and video, to express ideas, share experiences, and participate in communication (Masoumi & Bourbour, 2024; United Nations Committee on the Rights of the Child, 2021). Digital rules and risk prevention drew on the safety domain of DigComp 2.2, the balance between protection and participation emphasized in documents on children’s digital rights, and early childhood research on safe, responsible, and appropriate participation in digital environments. It emphasizes children’s emerging awareness of rules, safety, and risks in digital activities under adult guidance (L. Li et al., 2025; United Nations Committee on the Rights of the Child, 2021; Vuorikari et al., 2022).

#### 2.1.2. Item Generation

The theoretical analysis established a preliminary four-dimensional framework for young children’s digital literacy. Each dimension was then translated into specific behavioral indicators that were developmentally appropriate for young children aged 3–6 years and observable by parents. To identify behaviors that parents could observe at home, we conducted semistructured interviews with a purposively selected sample of eight experts in fields related to young children’s digital literacy (E1–E8) and 52 parents of children aged 3–6 years (P1–P52). The research team developed the interview guide around the four theoretical dimensions. Questions focused on specific behaviors and representative examples in young children’s everyday digital activities, including the contexts in which they used particular devices or content, the operations they could complete, how they understood and applied digital information, how they used digital media for expression and interaction, and how they followed rules and responded to potential risks. These accounts were used to identify behavioral indicators and generate initial items. All interviews were audio-recorded and transcribed after informed consent had been obtained. The formal interviews totaled 1686 min, with individual interviews lasting 21–38 min (*M* ≈ 28 min).

The interview data were analyzed thematically using NVivo 11.0. Initial analysis involved 56 interviews with seven experts and 49 parents. Three researchers with backgrounds in early childhood education first coded the interview material independently, compared their interpretations, and then continued coding collaboratively. Coding disagreements were discussed with reference to the original interview context, and each retained code required agreement from at least two researchers. Through constant comparison, overlapping codes were consolidated into observable behavioral indicators and mapped onto the preliminary theoretical dimensions. The remaining four interviews, comprising one expert and three parents, were subsequently analyzed to assess saturation. These four interviews yielded no additional indicators within the preliminary framework. The analysis generated 25 initial behavioral indicators: seven for digital operation and exploration, four for digital understanding and transfer, six for digital expression and interaction, and eight for digital rules and risk prevention. For example, within digital operation and exploration, Expert E1 noted that digital operation involves not only completing a single action but also carrying out multiple steps in sequence; this observation informed the indicator “can complete multistep digital operation tasks.” Within digital understanding and transfer, Parent P46 stated, “She learns letters and words from English-learning content. When she sees an apple in everyday life, she also says ‘apple.’” This account informed the indicator “can understand familiar digital content.” Within digital expression and interaction, Parent P3 stated, “She uses a mobile phone to record videos and then tells us what she recorded.” This account informed the indicator “can introduce their own digital creations, such as photographs or videos, to others.” Within digital rules and risk prevention, Expert E6 noted that young children should develop an emerging awareness of the physical discomfort that may accompany prolonged digital-device use; this observation informed the indicator “knows that using electronic devices for a long time can cause physical discomfort.”

#### 2.1.3. Expert Content Validation

Eleven university-based experts in early childhood education, all of whom held doctoral degrees, evaluated the content validity of the 25 initial items. Using a 4-point scale, the experts rated the relevance of each item to its proposed dimension and to the overall construct, where 1 indicated not relevant and 4 indicated highly relevant. The item-level content validity index (I-CVI) was calculated as the proportion of experts assigning a rating of 3 or 4, and 0.78 was used as the criterion for item retention. All items met this criterion except “When disagreements arise during digital collaboration, the child can try to negotiate with peers.” Its I-CVI was 0.73, it had relatively few sources and reference points in the interview data, and the experts considered peer negotiation too demanding for young children. The item was therefore removed. After the wording of several items had been revised in response to expert feedback, the final scale retained 24 items across four dimensions: 7 items for digital operation and exploration, 4 for digital understanding and transfer, 5 for digital expression and interaction, and 8 for digital rules and risk prevention (see Table 1). Scale-level content validity was assessed using the averaging method (S-CVI/Ave), calculated as the mean of the I-CVIs across the 24 retained items (Polit & Beck, 2006). The resulting S-CVI/Ave was 0.95, indicating high average expert agreement on item relevance.

### 2.2. Phase 2: Item Analysis and Exploratory Factor Analysis

#### 2.2.1. Participants and Procedure

Phase 2 examined item performance and explored the preliminary factor structure using a sample recruited from a district in Nantong, Jiangsu Province, China. The district is referred to as District C to protect participant confidentiality. The target population comprised kindergarten-enrolled children aged 3–6 years, with parents providing questionnaire reports about their children. With assistance from the district education authority, the researchers constructed a sampling frame from district-wide kindergarten enrollment records, using each child’s student registration number as a unique identifier. A computer program randomly selected 500 children from this frame, making the individual child the sampling unit. Parents of 412 selected children consented to participate and returned questionnaires, yielding a consent rate of 82.4%. After 14 questionnaires with evident patterned responding or excessive concentration on a single response option were excluded, 398 valid questionnaires remained, representing 96.6% of those returned. The children included 197 boys (49.5%) and 201 girls (50.5%); 247 lived in urban areas (62.1%) and 151 in rural areas (37.9%). The age groups comprised 130 children aged 3–4 years (32.7%), 140 aged 4–5 years (35.2%), and 128 aged 5–6 years (32.2%). This sample was used for item analysis and exploratory factor analysis.

Harman’s single-factor test was conducted as a preliminary assessment of common method bias in the exploratory sample (*N* = 398). An unrotated principal components analysis of the 24 digital literacy items yielded four components with eigenvalues greater than 1, jointly explaining 66.93% of the total variance. The first component accounted for 34.15% of the variance. No single component explained the majority of the item variance, although this finding does not rule out common method bias (Podsakoff et al., 2003).

#### 2.2.2. Item Analysis

Scores on all 24 items differed significantly between the high- and low-score groups, with critical ratios ranging from 9.82 to 14.65, indicating satisfactory item discrimination. Corrected item–total correlations ranged from 0.45 to 0.61 and exceeded 0.30, indicating acceptable consistency between each item and the total scale. Cronbach’s α for the total scale did not increase after the deletion of any item, suggesting that no item substantially weakened internal consistency. All 24 items developed in Phase 1 were therefore retained for exploratory factor analysis.

#### 2.2.3. Exploratory Factor Analysis

Exploratory factor analysis was conducted on the 24 items retained after item analysis. The Kaiser–Meyer–Olkin value was 0.93, and Bartlett’s test of sphericity was significant, χ^2^(276) = 5529.24, *p* < 0.001, indicating that the data were suitable for factor analysis. Parallel analysis supported retaining four factors. Principal axis factoring with direct oblimin rotation yielded a four-factor structure consistent with the theoretical framework. Together, the four factors explained 60.34% of the total item variance. Pattern coefficients on the intended factors ranged from 0.69 to 0.84, communalities ranged from 0.52 to 0.67, and all cross-loadings had absolute values below 0.30. Factor correlations ranged from 0.30 to 0.42, indicating that the factors were related but not highly overlapping (see Table 2).

### 2.3. Phase 3: Psychometric Evaluation

#### 2.3.1. Participants and Procedure

Phase 3 recruited an independent sample from a district in Wuhu, Anhui Province, China, referred to as District D to protect participant confidentiality. Recruiting a separate sample from another location allowed the factor structure identified in Phase 2 to be evaluated using independent data. The target population comprised kindergarten-enrolled children aged 3–6 years, with parents serving as informants. With assistance from the district education authority, the researchers constructed a sampling frame from district-wide kindergarten enrollment records and used student registration numbers to uniquely identify eligible children. A computer program randomly selected 700 children, whose parents were invited to participate. Parents of 622 children consented and returned questionnaires, yielding a consent rate of 88.9%. After 19 questionnaires with evident patterned responding or excessive concentration on a single response option were excluded, 603 valid questionnaires remained, representing 96.9% of those returned. The children included 298 boys (49.4%) and 305 girls (50.6%); 361 lived in urban areas (59.9%) and 242 in rural areas (40.1%). The age groups comprised 180 children aged 3–4 years (29.9%), 241 aged 4–5 years (40.0%), and 182 aged 5–6 years (30.2%). This sample was used for confirmatory factor analysis, reliability and construct validity assessments, and measurement invariance testing. It was also used in Phase 4 to examine associations between digital literacy and problematic media use.

#### 2.3.2. Confirmatory Factor Analysis

Based on the four-dimensional theoretical framework established in Phase 1 and the exploratory factor analysis results from Phase 2, confirmatory factor analysis of the 24 items was conducted in the independent validation sample to evaluate the hypothesized four-factor model. Four correlated latent variables were specified: digital operation and exploration, digital understanding and transfer, digital expression and interaction, and digital rules and risk prevention. Each item loaded only on its intended dimension. The model was estimated using weighted least squares with mean and variance adjustment (WLSMV) and showed good fit: χ^2^(246) = 810.609, χ^2^/*df* = 3.30, comparative fit index (CFI) = 0.981, Tucker–Lewis index (TLI) = 0.979, root mean square error of approximation (RMSEA) = 0.062, 90% confidence interval (CI) [0.057, 0.066], and standardized root mean square residual (SRMR) = 0.028. As shown in Table 2, standardized factor loadings ranged from 0.78 to 0.94 and were all significant (*p* < 0.001); item-level explained variance (*R*^2^) ranged from 0.61 to 0.89. Together, these results supported the proposed four-factor structure in the validation sample.

#### 2.3.3. Reliability and Construct Validity

Cronbach’s α was 0.96 for the total scale and 0.95, 0.91, 0.92, and 0.93 for digital operation and exploration, digital understanding and transfer, digital expression and interaction, and digital rules and risk prevention, respectively, indicating satisfactory internal consistency. Composite reliability (CR) values for the four dimensions were 0.97, 0.94, 0.96, and 0.95, and average variance extracted (AVE) values were 0.81, 0.79, 0.81, and 0.71, respectively. Correlations among the four dimensions ranged from 0.52 to 0.82. The square roots of the AVE values ranged from 0.84 to 0.90 and exceeded the corresponding interdimension correlations, meeting the Fornell–Larcker criterion; heterotrait–monotrait (HTMT) ratios ranged from 0.47 to 0.79, below the 0.85 threshold (Fornell & Larcker, 1981; Henseler et al., 2015). Digital understanding and transfer and digital expression and interaction had the highest correlation and HTMT ratio, at 0.82 and 0.79, respectively. Although these values met the stated criteria, the strong association between these two dimensions warrants further examination of their empirical distinctiveness.

#### 2.3.4. Measurement Invariance

Measurement invariance across child sex, place of residence, and age groups was examined by successively estimating configural, metric, and threshold (scalar) invariance models. Changes in CFI and RMSEA were used to assess whether adding equality constraints substantially worsened model fit (Chen, 2007; Cheung & Rensvold, 2002). A decrease in CFI of no more than 0.010 and an increase in RMSEA of no more than 0.015 were adopted as practical reference criteria (Chen, 2007). As shown in Table 3, the configural models showed acceptable fit across all three grouping variables. After equality constraints were imposed on factor loadings and item thresholds, CFI remained unchanged or increased, while RMSEA decreased. These findings provide preliminary support for measurement invariance across child sex, place of residence, and age groups, offering an initial measurement basis for comparing latent levels across these groups.

### 2.4. Phase 4: Validity Evidence Based on Relations with Problematic Media Use

Phase 4 used the validation sample from Phase 3 (*N* = 603) to examine associations between young children’s digital literacy scores and problematic media use. Digital literacy was assessed using the Digital Literacy Scale for Young Children developed in this study. Items were rated on a 5-point scale from 1 (very untrue) to 5 (very true), with higher scores indicating higher parent-reported digital literacy. Problematic media use was assessed using the Problematic Media Use Measure–Short Form (PMUM-SF) developed by Domoff et al. (2019). Parents rated their children’s media-use behavior during the previous month on a 5-point scale from 1 (never) to 5 (always), with higher scores indicating higher levels of problematic media use. In the present sample, Cronbach’s α for the PMUM-SF was 0.923, CR was 0.941, and AVE was 0.640. A one-factor confirmatory model estimated using WLSMV yielded an adjusted χ^2^(27) = 131.90, *p* < 0.001, robust CFI = 0.956, robust TLI = 0.942, and SRMR = 0.037.

Pearson correlations showed that the total digital literacy mean score and all four dimension scores were negatively correlated with problematic media use. The correlation for the total score was *r* = −0.58, and correlations for the four dimensions ranged from −0.39 to −0.56, all *ps* < 0.001.

#### Hierarchical Regression Analysis

Child sex, place of residence, and age group were entered in Step 1, followed by the mean scores for the four digital literacy dimensions in Step 2. Residual, collinearity, and influence diagnostics were examined (Ernst & Albers, 2017). Tolerance values for the four dimensions ranged from 0.37 to 0.58, and variance inflation factors ranged from 1.71 to 2.73, indicating no substantial multicollinearity. HC3 heteroscedasticity-consistent standard errors were used for statistical inference (Long & Ervin, 2000).

As shown in Table 4, the demographic variables explained little variance in problematic media use scores, *R*^2^ = 0.008, robust *F*(3, 599) = 1.41, *p* = 0.239. Adding the four digital literacy dimensions increased the explained variance to 36.5%, adjusted *R*^2^ = 0.357, Δ*R*^2^ = 0.356. Their joint contribution was significant, robust Wald *F*(4, 595) = 39.09, *p* < 0.001. In the final linear model, digital understanding and transfer (β = −0.265, *p* < 0.001), digital expression and interaction (β = −0.128, *p* = 0.021), and digital rules and risk prevention (β = −0.250, *p* < 0.001) were negatively associated with PMUM-SF scores. The coefficient for digital operation and exploration was not significant (β = −0.043, *p* = 0.307).

## 3. Discussion

Drawing on theoretical analysis and qualitative interviews, this study developed a parent-report digital literacy scale for children aged 3–6 years. Exploratory and confirmatory factor analyses supported four dimensions: digital operation and exploration, digital understanding and transfer, digital expression and interaction, and digital rules and risk prevention. In the adjusted regression model, understanding and transfer, expression and interaction, and rules and risk prevention were negatively associated with problematic media use, whereas the coefficient for operation and exploration was not significant.

The findings support conceptualizing young children’s digital literacy as a multidimensional developmental competency that extends beyond device operation. Previous research has often distinguished operational from cultural digital literacy, with the latter encompassing digital content understanding, meaning making, creative expression, and social interaction (Moon & Shin, 2025). Reviews also indicate that, with appropriate support, young children can develop foundational competencies in multimodal expression, reflective understanding, and active participation and can use digital technologies for collaboration, problem solving, and creative production (H. Li et al., 2026; Mecklenburg & Scheithauer, 2026). The scale translates these competencies into four sets of parent-report items.

Expert and parent interviews were used alongside the theoretical literature to identify specific behaviors observed in young children’s digital activities at home. Good practice in scale development recommends combining deductive sources, such as theoretical literature, with inductive sources, such as interviews, to improve construct coverage and item appropriateness (Boateng et al., 2018; Brod et al., 2009). In this study, expert judgments were used to clarify the construct domain and dimensional boundaries, whereas parents’ accounts informed the selection and wording of behaviors observable in everyday life. Young children’s digital practices are embedded in family routines, play, and interactions with adults and peers and are associated with home resources, parental use patterns, content selection, and media mediation (Ata-Aktürk & Akman, 2024; Papadopoulou et al., 2025).

The scale measures digital operation and exploration, digital understanding and transfer, digital expression and interaction, and digital rules and risk prevention as separate dimensions. This structure addresses both young children’s opportunities to use digital tools for expression, communication, and creation and their emerging awareness of boundaries, risks, and appropriate behavior. Digital education in the early years should incorporate safety rules and appropriate use into play and everyday activities in developmentally appropriate ways (L. Li et al., 2025).

The strong correlation between digital understanding and transfer and digital expression and interaction (*r* = 0.82) may partly reflect their joint involvement in digital activities. Research on digital storytelling illustrates how children interpret visual content while communicating meaning through drawings, speech, and gestures (Shengjergji et al., 2025). The two dimensions were designed to address different assessment targets. In this scale, the former emphasizes content interpretation, evaluation, and transfer to real-world activities, whereas the latter emphasizes expressing views, communicating, and collaborating. This distinction is consistent with research differentiating critical engagement with digital content from digital production (Masoumi & Bourbour, 2024). For example, explaining content preferences emphasizes the reasons underlying a judgment, whereas expressing views and feelings emphasizes communicating meaning. Although the items were designed to distinguish these aspects, the strong association between the dimensions calls for further examination of their empirical distinction.

Digital understanding and transfer was negatively associated with problematic media use after adjustment for demographic characteristics and the other dimensions. This dimension concerns whether young children understand digital content and connect the information they obtain with existing knowledge or real-world activities. Transfer of learning in digital environments is not automatic and varies with task characteristics, presentation contexts, and cognitive control (Mombo et al., 2024). Adult–child co-use has shown a small positive association with young children’s digital learning, and family members may use discussion and extensions into offline activities to connect digital content with everyday experience (Scott, 2022; Taylor et al., 2024). One possible explanation is that higher ratings of understanding and transfer reflect digital activities that are more closely connected to everyday experience and less centered on passive or repetitive engagement. Neither actual transfer performance nor these activity patterns were directly assessed, so this explanation remains to be tested.

Digital rules and risk prevention also showed a negative adjusted association with problematic media use. This dimension concerns following agreements, stopping at an appropriate time, maintaining boundaries for use, and responding to uncomfortable digital situations. These behaviors are closely related to the control difficulties and transition problems involved in problematic media use. Previous research found that higher problematic media use among children aged 3–5 years was associated with more frequent crying, pestering, and physical reactions when limits were imposed, as well as weaker emotional self-regulation (Emond et al., 2024). Self-regulation has generally shown weaker associations with overall screen duration but stronger associations with dysregulated digital engagement (Howard et al., 2025). Higher trajectories of screen use in early childhood have also been associated with higher problematic media use scores at age 5.5 years (Fitzpatrick et al., 2025). These findings suggest that digital rules and risk prevention may reflect aspects of young children’s regulation when stopping use, transitioning away from media, and maintaining activity boundaries.

Digital expression and interaction was also negatively associated with problematic media use after adjustment, whereas the coefficient for digital operation and exploration was not significant. For operation and exploration, the nonsignificant coefficient reflects its conditional association with problematic media use after accounting for demographic characteristics and the other dimensions. Even when young children frequently access digital devices, their activities may remain concentrated on viewing and gaming (Ata-Aktürk & Akman, 2024). Operational competency may provide a foundation for engaging with digital environments, whereas content understanding and behavioral regulation may relate to different aspects of that engagement. The adjusted coefficients therefore should not be interpreted as a ranking of the dimensions’ developmental importance.

Existing research has often emphasized the amount of media exposure while giving less attention to content, activity type, and context of use, although associations between screen use and child development may vary with purpose and social context (Harverson et al., 2025; Vasconcellos et al., 2025). For research on young children’s digital experiences, the scale can be used alongside indicators of screen duration, media content, and family support, with the invariance findings providing preliminary support for comparing latent levels across child sex, place of residence, and age groups.

The four-factor scale offers an initial research framework for assessing parent-reported digital competencies beyond device operation and examining their links to family support and media engagement in early childhood. Evidence remains insufficient for individual classification or treatment planning. Low scores should not be interpreted as developmental deficits or a need for greater digital exposure. Adults can support children through joint engagement, discussion of digital content, and clear boundaries for use, while retaining primary responsibility for media selection, supervision, and safety.

### Limitations and Future Research

First, the correspondence between parent-reported scores and children’s directly observed digital competence remains to be established. Low ratings may reflect limited activity opportunities or parental observation rather than low competence. Adult assistance, language ability, and general self-regulation may also influence ratings. Future research should use cognitive interviews to clarify how parents interpret items and select responses. Standardized performance tasks that document adult assistance could provide external criteria for validating parent-reported scores.

Second, concurrent parent reports of digital literacy and problematic media use may be affected by common method bias. Parents’ overall impressions and socially desirable responding may inflate or attenuate the observed associations. Harman’s single-factor test covered only digital literacy items in the exploratory sample and cannot rule out such bias in these associations (Podsakoff et al., 2003). Future studies should incorporate teacher ratings, behavioral observations, and objective digital records to obtain evidence across informants and methods.

Third, the cross-sectional design cannot establish temporal ordering or causality, leaving reverse associations and unmeasured confounding unresolved. Items concerning rules and stopping device use may also overlap with aspects of problematic media use, potentially contributing to the observed associations. Longitudinal or intervention studies are needed to clarify the direction of these relationships, alongside closer examination of item content to assess potential overlap.

Finally, broader applicability requires validation beyond the two locations in eastern China represented in this study. The current measurement invariance results cannot substitute for cross-language and cross-cultural validation. Further research should examine test–retest reliability, predictive validity, and interrater agreement, and assess the feasibility of developing a short form. At present, the scale remains an initial parent-report measure of young children’s digital competencies within the contexts represented by these samples, and the findings do not establish age-based developmental norms.

## Figures and Tables

**Table 1:** Retained Item Development and Content Validity Evaluation.

Dimension	Digital Literacy Indicator	NS	RP	I-CVI
Digital operation and exploration	DO1 Knows the main function of common operation buttons (e.g., “×” means close).	20	22	1.00
	DO2 Can complete simple operations by tapping and swiping.	40	50	1.00
	DO3 Can complete multistep digital operation tasks (e.g., use several steps to find a desired television program).	34	51	0.91
	DO4 Can use search functions to access digital content (e.g., songs or stories).	37	47	0.91
	DO5 Can save their own digital creations.	17	17	0.82
	DO6 Can try different approaches when an operation is unsuccessful.	27	35	1.00
	DO7 Can close or skip disruptive content such as advertisements and pop-up windows.	23	24	1.00
Digital understanding and transfer	DU1 Can understand familiar digital content (e.g., understand videos they commonly watch).	27	35	1.00
	DU2 Can distinguish animated content from real life.	18	21	0.91
	DU3 Can explain why they like or dislike particular digital content.	31	38	1.00
	DU4 Can begin to apply knowledge or methods learned from digital content to real-world activities.	34	43	1.00
Digital expression and interaction	DE1 Can express views and feelings about digital content or digital operations.	31	42	1.00
	DE2 Can respond to others using images, audio recordings, or emojis.	23	26	0.91
	DE3 Can communicate with others through voice or video.	46	54	1.00
	DE4 Can introduce their own digital creations, such as photographs or videos, to others.	23	27	1.00
	DE5 Can collaborate with others to complete a digital task (e.g., play Gomoku together on a tablet).	15	17	0.82
Digital rules and risk prevention	DR1 Knows that using electronic devices for a long time can cause physical discomfort.	29	34	0.91
	DR2 Can maintain an appropriate viewing distance and a healthy posture during use.	32	35	1.00
	DR3 Can follow rules for using electronic devices.	27	31	1.00
	DR4 Can stop using an electronic device at an agreed time or when prompted by a timer.	26	35	0.91
	DR5 Asks permission before using another person’s electronic device.	26	28	0.91
	DR6 Can take turns as agreed when sharing an electronic device with others.	17	17	0.82
	DR7 Does not disclose personal information to others without permission (e.g., device passwords or personal details).	9	9	1.00
	DR8 Stops the activity and seeks adult help when encountering an uncomfortable digital situation.	32	43	1.00

Note. NS = number of sources, indicating the number of participants who mentioned the indicator; RP = reference points, indicating the number of times the indicator was coded in the interview data; I-CVI = item-level content validity index.

**Table 2:** Exploratory and Confirmatory Factor Analysis Results for the Digital Literacy Scale for Young Children.

Dimension	Item	h^2^	EFA λ	CFA λ
Digital operation and exploration	DO1	0.54	0.73	0.94
	DO2	0.63	0.80	0.94
	DO3	0.63	0.78	0.88
	DO4	0.55	0.74	0.85
	DO5	0.52	0.71	0.89
	DO6	0.58	0.75	0.89
	DO7	0.60	0.77	0.91
Digital understanding and transfer	DU1	0.55	0.72	0.89
	DU2	0.66	0.80	0.84
	DU3	0.67	0.84	0.93
	DU4	0.54	0.72	0.90
Digital expression and interaction	DE1	0.57	0.71	0.89
	DE2	0.62	0.81	0.90
	DE3	0.62	0.81	0.93
	DE4	0.57	0.73	0.94
	DE5	0.58	0.69	0.83
Digital rules and risk prevention	DR1	0.55	0.76	0.84
	DR2	0.65	0.79	0.80
	DR3	0.66	0.81	0.87
	DR4	0.65	0.80	0.86
Digital rules and risk prevention (continued)	DR5	0.65	0.78	0.88
	DR6	0.62	0.76	0.89
	DR7	0.64	0.80	0.78
	DR8	0.63	0.81	0.81

Note. h^2^ = communality, or the proportion of item variance explained by the extracted common factors; λ = factor loading; EFA = exploratory factor analysis; CFA = confirmatory factor analysis. EFA coefficients are pattern coefficients after direct oblimin rotation in the exploratory sample (*N* = 398). CFA coefficients are standardized factor loadings in the validation sample (*N* = 603).

**Table 3:** Measurement Invariance Results.

Grouping Variable	Model	χ^2^	*df*	CFI	TLI	RMSEA	ΔCFI	ΔRMSEA
Child sex	Configural invariance	1157.542	492	0.979	0.977	0.067	—	—
	Metric invariance	1162.153	512	0.980	0.978	0.065	0.001	−0.002
	Threshold (scalar) invariance	1221.755	580	0.980	0.981	0.061	0.000	−0.004
Place of residence	Configural invariance	1079.050	492	0.981	0.979	0.063	—	—
	Metric invariance	1085.239	512	0.982	0.980	0.061	0.001	−0.002
	Threshold (scalar) invariance	1122.764	580	0.983	0.983	0.056	0.001	−0.005
Age group	Configural invariance	1343.229	738	0.982	0.980	0.064	—	—
	Metric invariance	1354.504	778	0.983	0.982	0.061	0.001	−0.003
	Threshold (scalar) invariance	1429.553	914	0.985	0.986	0.053	0.002	−0.008

Note. ΔCFI and ΔRMSEA were calculated by subtracting the fit index of the preceding, less constrained model from that of the more constrained model. The metric model was compared with the configural model, and the threshold (scalar) model with the metric model. Practical reference criteria were ΔCFI ≥ −0.010 and ΔRMSEA ≤ 0.015 (Chen, 2007). CFI = comparative fit index; TLI = Tucker–Lewis index; RMSEA = root mean square error of approximation.

**Table 4:** Hierarchical Regression of Problematic Media Use on Digital Literacy Dimensions with HC3 Standard Errors.

Model	Variable	*B*	*SE* (HC3)	β	*p*
Step 1	Child sex	0.095	0.061	0.063	0.120
	Place of residence	0.062	0.064	0.040	0.338
	Age group	−0.054	0.040	−0.056	0.176
Step 2	Child sex	0.083	0.050	0.055	0.098
	Place of residence	0.019	0.053	0.012	0.723
	Age group	−0.003	0.033	−0.004	0.919
	Digital operation and exploration	−0.032	0.032	−0.043	0.307
	Digital understanding and transfer	−0.246	0.060	−0.265	<0.001
	Digital expression and interaction	−0.113	0.049	−0.128	0.021
	Digital rules and risk prevention	−0.244	0.054	−0.250	<0.001

Note. The dependent variable was the PMUM-SF mean score. *B* = unstandardized regression coefficient; *SE* = standard error; β = standardized regression coefficient. Standard errors and two-sided *p* values use the HC3 estimator. Both models included an intercept, which is omitted from the table. Child sex: boy = 1, girl = 2; place of residence: urban = 1, rural = 2; age group: 3–4 years = 1, 4–5 years = 2, 5–6 years = 3. Step 1: *R*^2^ = 0.008, adjusted *R*^2^ = 0.003, robust *F*(3, 599) = 1.41, *p* = 0.239. Step 2: *R*^2^ = 0.365, adjusted *R*^2^ = 0.357, Δ*R*^2^ = 0.356; robust Wald test of the four added dimensions: *F*(4, 595) = 39.09, *p* < 0.001.

## Data Availability

The de-identified datasets generated and analyzed in this study are available from the corresponding author upon reasonable request, subject to applicable ethical and data protection requirements.
